# Discrete-Time Adaptive Control for Three-Phase PWM Rectifier

**DOI:** 10.3390/s24103010

**Published:** 2024-05-09

**Authors:** Bo Hou, Jiayan Qi, Huan Li

**Affiliations:** School of Electrical Engineering, Shaanxi University of Technology, Hanzhong 723001, China; qijiayan@snut.edu.cn (J.Q.); lihuan@snut.edu.cn (H.L.)

**Keywords:** three-phase PWM rectifier, discrete-time adaptive control, inductance parameter mismatches, load disturbance, discrete-time feedback linearization control

## Abstract

This paper proposes a dual-loop discrete-time adaptive control (DDAC) method for three-phase PWM rectifiers, which considers inductance-parameter-mismatched and DC load disturbances. A discrete-time model of the three-phase PWM rectifier is established using the forward Euler discretization method, and a dual-loop discrete-time feedback linearization control (DDFLC) is given. Based on the DDFLC, the DDAC is designed. Firstly, an adaptive inductance disturbance observer (AIDO) based on the gradient descent method is proposed in the current control loop. The AIDO is used to estimate lump disturbances caused by mismatched inductance parameters and then compensate for these disturbances in the current controller, ensuring its strong robustness to inductance parameters. Secondly, a load parameter adaptive law (LPAL) based on the discrete-time Lyapunov theory is proposed for the voltage control loop. The LPAL estimates the DC load parameter in real time and subsequently adjusts it in the voltage controller, achieving DC load adaptability. Finally, simulation and experimental results show that the DDAC exhibits better steady and dynamic performances, less current harmonic content than the DDFLC and the dual-loop discrete-time PI control (DDPIC), and a stronger robustness to inductance parameters and DC load disturbances.

## 1. Introduction

**Figure 1:**
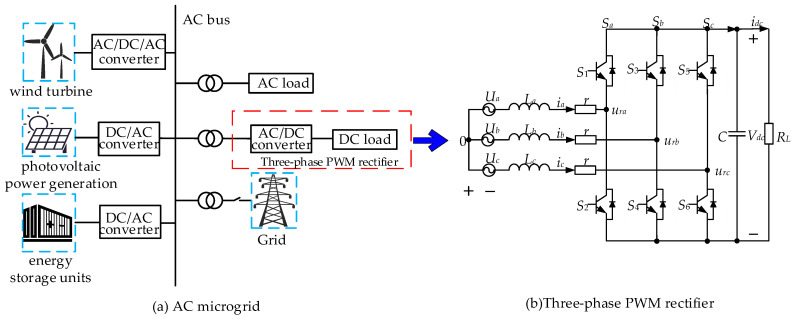
Rectifier system in an AC microgrid.

Microgrids (MG) are essential components of modern power systems, offering various benefits including environmental friendliness, economic viability, flexibility, controllability, and high-power electronics [1]. As illustrated in Figure 1, rectifiers establish connections between AC buses and DC loads. In this context, the primary control objectives of rectifiers are to maintain a stable DC bus voltage for the DC load, operate at a unity power factor, and draw grid current with minimal harmonic distortion. However, PWM rectifiers are inherently nonlinear, multivariable, and coupled systems [2], and their control performances are susceptible to practical disturbances such as DC load disturbances and mismatched parameters (parameter disturbances). Therefore, anti-disturbance control strategies for rectifiers have garnered significant attention in recent years [3,4,5].

Currently, three-phase PWM rectifiers typically use a dual-loop control structure consisting of a voltage outer loop and a current inner loop [2]. Linear proportional–integral (PI) controllers are commonly used in this structure because of their simple structure and ease of engineering implementation. However, PI controllers have a relatively slow dynamic response. Moreover, since PI controllers are designed with a bounded operating range, their anti-disturbance performance degrades when the system encounters a large disturbance. Consequently, scholars have proposed various control methods to enhance the rectifier’s resilience to disturbances. These methods mainly include backstepping control (BSC) [6], passivity-based control (PBC) [7,8], sliding mode control (SMC) [9,10], and adaptive control [11,12,13,14].

Of these control methods for rectifiers, adaptive control is a powerful control method, playing a leading role in addressing the global stability problems of nonlinear systems subject to parameter uncertainty and disturbance. Reference [11] introduces an adaptive BSC to compensate for the inherent nonlinearities and uncertainties in the rectifier. On this basis, reference [12] presents an improved adaptive backstepping sliding mode control, which enhances the global stability of the adaptive BSC by incorporating error compensation and SMC. Reference [13] proposes a robust adaptive control for a three-phase PFC converter. This method utilizes a model reference adaptive control for the voltage outer loop to adapt to loads and capacitor variations. Simultaneously, SMC is used to strengthen the robustness of the current controller. In [14], an efficient adaptive controller is established in the voltage outer loop to improve the controller’s ability to regulate DC bus voltage in the presence of external disturbances, and H∞ controllers are applied in the current loop. References [11,12,13,14] employ adaptive control to substantially enhance rectifier performance from multiple perspectives. However, the methods in [11,12,13,14] are designed in the continuous-time domain and are not directly applicable to a microprocessor using a digital controller. In recent years, with the increasing speed and decreasing cost of microprocessors, controller design utilizing discrete-time control has become a research hotspot in power electronics [15]. Additionally, it is well known that discrete-time systems, rather than continuous-time systems, are widely regarded as being closer to describing a real controlled system [16].

Currently, discrete-time adaptive control is widely used in power electronic systems [17,18,19,20]. Reference [17] introduces a discrete-time model reference adaptive control method to reduce the number of sensors and improve robustness against unmodeled dynamics and sinusoidal disturbances in an LCL grid-connected inverter. Reference [18] proposes a discrete-time model reference adaptive controller based on adaptive super-twisting sliding mode control, effectively suppressing the 5th, 7th, 11th, and 13th current harmonic components. Reference [19] introduces a new discrete-time direct robust adaptive PI controller featuring fast current tracking, robustness to disturbances and grid inductance variations, and global stability. References [17,18,19] demonstrate the feasibility and effectiveness of employing discrete-time adaptive control in power electronic systems. However, to the best of our knowledge, most dual-loop adaptive controller methods for three-phase PWM rectifiers are formulated in the continuous-time domain [21,22]; there are no reports on dual-loop discrete-time adaptive controller methods for rectifiers.

This paper proposes a dual-loop discrete-time adaptive control (DDAC) method for three-phase PWM rectifiers, addressing inductance-parameter-mismatched and DC load disturbances. The main contributions of this work include the following:

1. An adaptive inductance disturbance observer (AIDO) is developed in the current control loop using the gradient descent method, ensuring its strong robustness and adaptability to mismatched inductance parameters.
2. A load parameter adaptive law (LPAL) is developed in the DC bus voltage control loop using the discrete-time Lyapunov stability theory, improving the DC load disturbances rejection ability of the DC bus voltage regulator.
3. Comparison experiments are conducted between the DDAC, dual-loop discrete-time feedback linearization control (DDFLC), and dual-loop discrete-time PI control (DDPIC) in a real three-phase PWM rectifier, thereby verifying the superiority of the DDAC.

The remainder of this paper is organized as follows: Firstly, Section 2 briefly introduces a discrete-time model of three-phase PWM rectifiers. Secondly, the shortcomings of the DDFLC are discussed and analyzed in Section 3. After that, in Section 4, the design and analysis of the DDAC are presented in detail. Then, Section 5 presents the simulation and experimental results of the proposed DDAC, which are compared with those of the DDFLC and the DDPIC to verify its effectiveness and advantages. Finally, some conclusions are given in Section 6.

## 2. Discrete-Time Model of Three-Phase PWM Rectifiers

The AC MG is depicted in Figure 1a. Since the energy storage unit can stabilize the AC bus, the AC bus is considered an ideal AC source in this paper. The circuit of a three-phase PWM rectifier is shown in Figure 1b. *U_a_*, *U_b_*, and *U_c_* are the three-phase grid voltages; *V_dc_* is the DC bus voltage; *i_a_*, *i_b_*, and *i_c_* are the three-phase grid currents; *L* is the inductance; *r* is the equivalent resistance of the inductance; *C* is the filter capacitance; and *R_L_* is the DC load. From Figure 1b, the three-phase PWM rectifier dq model is modeled as follows [23].

(1) $$\left\{\begin{array}{l} L\frac{di_{d}}{dt}=U_{d}-ri_{d}+\omega Li_{q}-u_{rd}\\ L\frac{di_{q}}{dt}=U_{q}-ri_{q}-\omega Li_{d}-u_{rq}\\ C\frac{dV_{dc}}{dt}=\frac{3}{2}(S_{d}i_{d}+S_{q}i_{q})-\frac{V_{dc}}{R_{L}} \end{array}\right.$$

where *ω* represents the voltage angular frequency, *U_d_* and *U_q_* are the active and reactive voltage, *i_d_* and *i_q_* are the active and reactive current, *S_d_* and *S_q_* are the d-axis and q-axis switching components, and *u_rd_* = *S_d_V_dc_* and *u_rq_* = *S_q_V_dc_* represent the control inputs.

Considering the fact that *L* and *r* change in a certain range during rectifier operation [24,25], the dq model can be rewritten as follows:

(2) $$\left\{\begin{array}{l} L_{0}\frac{di_{d}}{dt}=U_{d}-r_{0}i_{d}+\omega L_{0}i_{q}-u_{rd}-f_{d}\\ L_{0}\frac{di_{q}}{dt}=U_{q}-r_{0}i_{q}-\omega L_{0}i_{d}-u_{rq}-f_{q}\\ C\frac{dV_{dc}}{dt}=\frac{3}{2}(S_{d}i_{d}+S_{q}i_{q})-\frac{V_{dc}}{R_{L}} \end{array}\right.$$

where *f_d_* and *f_q_* denote the inductance parameter disturbances induced by *L* and *r*.

(3) $$\left\{\begin{array}{l} f_{d}=\Delta ri_{d}+\Delta L\frac{di_{d}}{dt}-\Delta L\omega i_{q}\\ f_{q}=\Delta ri_{q}+\Delta L\frac{di_{q}}{dt}+\Delta L\omega i_{d} \end{array}\right.$$

where *L* = *L*_0_ + Δ*L*. *r* = *r*_0_ + Δ*r*. The forward Euler discretization method is used to discretize Equation (2), which yields

(4) $$\left\{\begin{array}{l} L_{0}\frac{i_{d}(k+1)-i_{d}(k)}{T_{s}}=U_{d}(k)-r_{0}i_{d}(k)+\omega L_{0}i_{q}(k)-u_{rd}(k)-f_{d}(k)\\ L_{0}\frac{i_{q}(k+1)-i_{q}(k)}{T_{s}}=U_{q}(k)-r_{0}i_{q}(k)-\omega L_{0}i_{d}(k)-u_{rq}(k)-f_{q}(k)\\ C\frac{V_{dc}(k+1)-V_{dc}(k)}{T_{s}}=\frac{3}{2}\left(S_{d}(k)i_{d}(k)+S_{q}(k)i_{q}(k)\right)-\frac{V_{dc}(k)}{R_{L}} \end{array}\right.$$

where *T_s_* denotes the sampling time and $\left\{\begin{array}{l} f_{d}(k)=\Delta ri_{d}(k)+\Delta L\frac{i_{d}(k+1)-i_{d}(k)}{T_{s}}-\Delta L\omega i_{q}(k)\\ f_{q}(k)=\Delta ri_{q}(k)+\Delta L\frac{i_{q}(k+1)-i_{q}(k)}{T_{s}}+\Delta L\omega i_{d}(k) \end{array}\right.$.

Since the bandwidth of the current loop is considerably larger than that of the voltage loop, it can be assumed that *i_d_* (*k*) = i_d_^*^(*k*) and *i_q_*(*k*) = i_q_^*^(*k*) in a steady state. i_d_^*^(*k*) and i_q_^*^(*k*) are the d-axis and q-axis reference currents, respectively. Considering that the three-phase PWM rectifier normally operates at a unity power factor, i_q_^*^(*k*) = 0. Consequently, the discrete-time model of the rectifier can be obtained from Equation (4).

(5) $$\left\{\begin{array}{l} L_{0}\frac{i_{d}(k+1)-i_{d}(k)}{T_{s}}=U_{d}(k)-r_{0}i_{d}(k)+\omega L_{0}i_{q}(k)-u_{rd}(k)-f_{d}(k)\\ L_{0}\frac{i_{q}(k+1)-i_{q}(k)}{T_{s}}=U_{q}(k)-r_{0}i_{q}(k)-\omega L_{0}i_{d}(k)-u_{rq}(k)-f_{q}(k)\\ C\frac{V_{dc}(k+1)-V_{dc}(k)}{T_{s}}=u_{rdc}(k)-\xi (k)V_{dc}(k) \end{array}\right.$$

where $u_{rdc}(k)=\frac{3}{2}S_{d}(k){i_{d}}^{*}(k)$, $\xi (k)=\frac{1}{R_{L}}$.

## 3. Design of the DDFLC

For the purpose of designing the controller efficiently and conveniently, the control objectives of these controllers are listed as follows:

1. In the current control loop, tracking the respective references of the active current *i_d_* and reactive current *i_q_* is required. In this control module, the reference of the active current i_d_^*^ is calculated based on the DC bus voltage control loop, and the reference of the reactive current i_q_^*^ is set to 0.
2. In the DC bus voltage control loop, the DC bus voltage *V_dc_* must be controlled according to reference V_dc_^*^ when the system achieves a stable state.

Based on the above control objectives, current tracking errors and the voltage tracking error are defined as follows:

(6) $$\left\{\begin{array}{l} e_{id}(k)=i_{d}(k)-{i_{d}}^{*}(k)\\ e_{iq}(k)=i_{q}(k)-{i_{q}}^{*}(k)\\ e_{u}(k)=V_{dc}(k)-{V_{dc}}^{*}(k) \end{array}\right.$$

where V_dc_^*^ is the reference DC bus voltage. Combining Equation (6) and the DFLC theory [15], we can obtain the current and voltage controllers, shown as follows:

(7) $$\left\{\begin{array}{l} u_{rd}(k)=U_{d}(k)+\omega L_{0}i_{q}(k)-r_{0}i_{d}(k)-L_{0}\left[\frac{{i_{d}}^{*}(k+1)-{i_{d}}^{*}(k)}{T_{s}}-k_{d}e_{id}(k)\right]\\ u_{rq}(k)=U_{q}(k)-\omega L_{0}i_{d}(k)-r_{0}i_{q}(k)-L_{0}\left[\frac{{i_{q}}^{*}(k+1)-{i_{q}}^{*}(k)}{T_{s}}-k_{q}e_{iq}(k)\right]\\ u_{rdc}(k)=C\left[\frac{{V_{dc}}^{*}(k+1)-{V_{dc}}^{*}(k)}{T_{s}}-k_{vdc}e_{u}(k)\right] \end{array}\right.$$

where *k_d_* and *k_q_* are the control parameters for the current loop and *k_vdc_* is a control parameter for the voltage loop, *k_d_* > 0, *k_q_* > 0, *k_Vdc_* > 0. By substituting Equation (7) into Equation (5), it can be found that

(8) $$\left\{\begin{array}{l} e_{id}(k+1)=e_{id}(k)-T_{s}k_{d}e_{id}(k)-\frac{T_{s}}{L_{0}}f_{d}(k)\\ e_{iq}(k+1)=e_{iq}(k)-T_{s}k_{q}e_{iq}(k)-\frac{T_{s}}{L_{0}}f_{q}(k)\\ e_{u}(k+1)=e_{u}(k)-T_{s}k_{vdc}e_{u}(k)-\frac{T_{s}}{C}\frac{V_{dc}(k)}{R_{L}} \end{array}\right.$$

**Lemma** **1** [26]**.** For the system z(k+1)=z(k)−lz(k)+g(k), if $\left|l\right|<1$ and $\left|g(k)\right|<\gamma ,\gamma >0$, then it follows that z(k) is always bounded. There exists a finite number K* > 0 such that $\left|z(k)\right|<\frac{\gamma }{\left|l\right|},\forall k>K^{*}$.

**Assumption** **1.** *The disturbances f_d_(k), f_q_(k), and ξ(k) are bounded, and they satisfy $\left|f_{d}(k)\right|<M,\left|f_{q}(k)\right|<N,\left|\xi (k)\right|<G$, M > 0, N > 0, G > 0.*

In accordance with the stipulations of Lemma 1, it can be demonstrated that the control parameters must satisfy the following conditions:

(9) $$0<k_{d}<\frac{1}{T_{s}},0<k_{q}<\frac{1}{T_{s}},0<k_{vdc}<\frac{1}{T_{s}}$$

then the steady state errors are bounded and satisfy

(10) $$\left|e_{id}(k)\right|\leq \frac{M}{L_{0}k_{d}},\left|e_{iq}(k)\right|\leq \frac{N}{L_{0}k_{q}},\left|e_{u}(k)\right|\leq \frac{G}{Ck_{vdc}}$$

From Equation (10), it can be seen that *e_id_*(*k*), *e_iq_*(*k*), and *e_u_*(*k*) will increase with the increments of *M*, *N*, and *G*, and that increasing *k_d_*, *k_q_*, and *k_vdc_* aids in decreasing tracking errors. However, *k_d_*, *k_q_*, and *k_vdc_* cannot be too large due to the fact that excessive gain will lead to system instability [15]. Therefore, with the DDFLC, it is challenging to achieve no tracking error in the presence of mismatched parameters or load conditions. To address this issue, this paper proposes the DDAC method for three-phase PWM rectifiers.

## 4. Design of the DDAC

**Figure 2:**
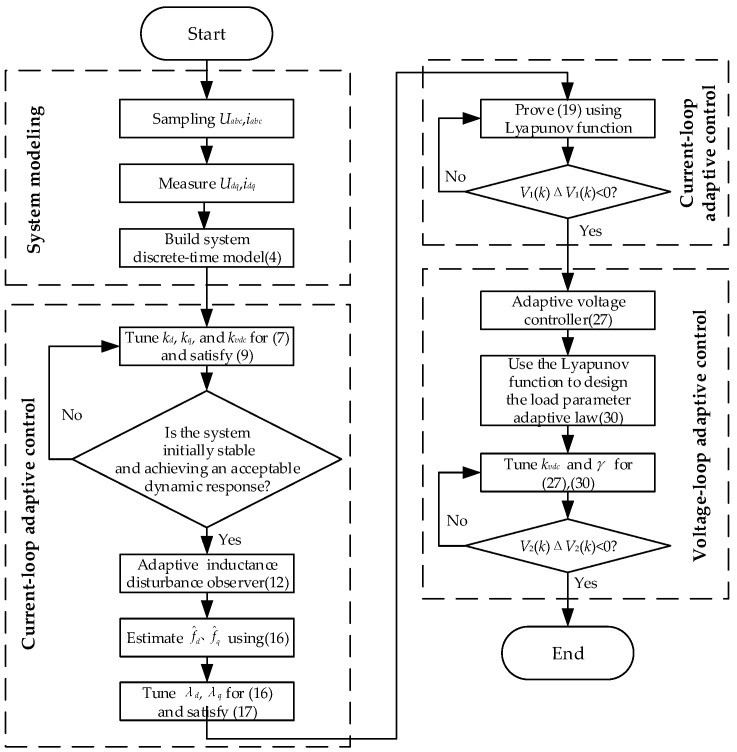
Flow diagram of the controller design.

Based on the DDFLC, this section proposes an AIDO for the current inner loop and an LPAL for the voltage outer loop. The AIDO, designed using the gradient descent method, estimates mismatched inductance parameter disturbances and compensates for them in the current controller, ensuring a strong robustness to inductance parameters. The LPAL, designed based on the discrete-time Lyapunov stability theory, estimates the DC load and adjusts it within the voltage controller, thereby achieving DC load adaptability. The design process is as follows, and a flow diagram of the DDAC’s design is shown in Figure 2.

### 4.1. Adaptive Controller for the Current Loop

According to Equation (5), the current discrete-time model can be expressed as

(11) $$i_{s}(k+1)=Ai_{s}(k)+B[v_{s}(k)-u_{rs}(k)-f_{s}(k)]$$

where $i_{s}(k)=\left(\begin{array}{l} i_{d}(k)\\ i_{q}(k) \end{array}\right)$, $v_{s}(k)=\left(\begin{array}{l} U_{d}(k)+\omega L_{0}i_{q}(k)\\ U_{q}(k)-\omega L_{0}i_{d}(k) \end{array}\right)$, $u_{rs}(k)=\left(\begin{array}{l} u_{rd}(k)\\ u_{rq}(k) \end{array}\right)$, $f_{s}(k)=\left(\begin{array}{l} f_{d}(k)\\ f_{q}(k) \end{array}\right)$, $A=1-\frac{r_{0}T_{s}}{L_{0}}$, $B=\frac{T_{s}}{L_{0}}$.

To estimate *f_s_*(*k*), a current adaptive observer with an input–output relationship is designed, as shown in Equation (12).

(12) $${\hat{i}}_{s}(k+1)=Ai_{s}(k)+B[v_{s}(k)-u_{rs}(k)-{\hat{f}}_{s}(k)]$$

where ${\hat{i}}_{s}(k)$ is the estimated value of *i_s_*(*k*) and ${\hat{f}}_{s}(k)$ is the estimated value of *f_s_*(*k*).

The disturbance estimation error *e_s_*(*k*) and ${\tilde{f}}_{s}(k)$ are defined as

(13) $$\begin{array}{l} e_{s}(k)=\left(\begin{array}{l} e_{d}(k)\\ e_{q}(k) \end{array}\right)=\left(\begin{array}{l} i_{d}(k)-{\hat{i}}_{d}(k)\\ i_{q}(k)-{\hat{i}}_{q}(k) \end{array}\right)\\ {\tilde{f}}_{s}(k)=\left(\begin{array}{l} f_{d}(k)\\ f_{q}(k) \end{array}\right)=\left(\begin{array}{l} f_{d}(k)-{\hat{f}}_{d}(k)\\ f_{q}(k)-{\hat{f}}_{q}(k) \end{array}\right) \end{array}$$

The gradient descent method is employed to design the AIDO. The gradient descent method is a local parameter optimization approach that assumes that parameters should be updated in a way that minimizes estimation errors [20]. Therefore, the following estimation error functions are considered as candidates:

(14) $$E_{s}(k)=\left(\begin{array}{l} E_{d}(k)\\ E_{q}(k) \end{array}\right)=\left(\begin{array}{l} \frac{1}{2}{e_{d}}^{2}(k)\\ \frac{1}{2}{e_{q}}^{2}(k) \end{array}\right)$$

Combining Equations (11)–(14), the following Jacobian matrix *J* can be obtained.

(15) $$J=\frac{\partial E_{s}(k)}{\partial {\hat{f}}_{s}(k)}=Be_{s}(k)$$

In accordance with the gradient descent idea [20], ${\hat{f}}_{s}(k)$ should change in the direction of the negative gradient. Combining this with Equation (15), this paper proposes an AIDO as follows:

(16) $${\hat{f}}_{s}(k+1)={\hat{f}}_{s}(k)+\Delta {\hat{f}}_{s}(k)={\hat{f}}_{s}(k)-\lambda Be_{s}(k)$$

where *λ_d_*, *λ_q_* are adaptive gain, and they satisfy

(17) $$0<\lambda_{d}<\frac{2}{B^{2}},0<\lambda_{q}<\frac{2}{B^{2}}$$

Based on the concept of feed-forward compensation, we develop current controllers as follows:

(18) $$u_{rs}(k)=\left(\begin{array}{l} u_{rd}(k)\\ u_{rd}(k) \end{array}\right)=\left(\begin{array}{l} U_{d}(k)+\omega L_{0}i_{q}(k)-{\hat{f}}_{d}(k)-r_{0}i_{d}(k)-L_{0}\left[\frac{{i_{d}}^{*}(k+1)-{i_{d}}^{*}(k)}{T_{s}}-k_{d}e_{id}(k)\right]\\ U_{q}(k)-\omega L_{0}i_{d}(k)-{\hat{f}}_{q}(k)-r_{0}i_{q}(k)-L_{0}\left[\frac{{i_{q}}^{*}(k+1)-{i_{q}}^{*}(k)}{T_{s}}-k_{q}e_{iq}(k)\right] \end{array}\right)$$

To prove the stability of the current adaptive observer, we define the Lyapunov function as

(19) $$V_{1}(k)=\frac{1}{2}{e_{s}}^{2}(k)$$

**Assumption** **2.** *The disturbance f_s_(k) is slow time-varying, it satisfies f_s_(k) = f_s_(k + 1).*

Combining Equations (11), (12), and (16), and Assumption 2, it obtains

(20) $$\Delta e_{s}(k)=e_{s}(k+1)-e_{s}(k)=-\lambda B^{2}e_{s}(k)$$

From Equations (19) and (20), we obtain

(21) $$\begin{array}{c} \Delta V_{1}(k)=V_{1}(k+1)-V_{1}(k)=\frac{1}{2}\left[{e_{s}}^{2}(k+1)-{e_{s}}^{2}(k)\right]\\ =\left(-\lambda B^{2}{e_{s}}^{2}(k)+\frac{1}{2}\lambda^{2}B^{4}{e_{s}}^{2}(k)\right) \end{array}$$

From Equation (17), this can be obtained as

(22) $$-\lambda B^{2}{e_{s}}^{2}(k)+\frac{1}{2}\lambda^{2}B^{4}{e_{s}}^{2}(k)<0$$

Therefore Δ*V*_1_ < 0. According to the discrete Lyapunov stability condition [27]

(23) $$V_{1}(k)\Delta V_{1}(k)<0$$

From Equation (23), it is evident that *e_s_*(*k*) will converge to zero. There is *e_s_*(*k*) = −*B*${\tilde{f}}_{s}(k)$, thus ${\tilde{f}}_{s}(k)$ will converge to zero. Consequently, it is reasonable to assume that there exists a finite number K*_1_ > 0 such that

(24) $$\left|{\tilde{f}}_{d}(k)\right|\leq M_{1},\left|{\tilde{f}}_{q}(k)\right|\leq N_{1},\forall k\geq K_{1}^{*}$$

where 0 < *M*_1_ << *M*, 0 < *N*_1_ << *N*. Substituting Equation (18) into Equation (5), it can be found that

(25) $$\left\{\begin{array}{l} e_{id}(k+1)=e_{id}(k)-T_{s}k_{d}e_{id}(k)-\frac{T_{s}}{L_{0}}{\tilde{f}}_{d}(k)\\ e_{iq}(k+1)=e_{iq}(k)-T_{s}k_{q}e_{iq}(k)-\frac{T_{s}}{L_{0}}{\tilde{f}}_{q}(k) \end{array}\right.$$

From Equations (24) and (25), and Lemma 1, we obtain

(26) $$\left|e_{id}(k)\right|\leq \frac{M_{1}}{L_{0}k_{d}}<<\frac{M}{L_{0}k_{d}},\left|e_{iq}(k)\right|\leq \frac{N_{1}}{L_{0}k_{q}}<<\frac{N}{L_{0}k_{q}},\forall k\geq K_{1}^{*}$$

It can be seen that, with the same control parameters *k_d_* and *k_q_*, the proposed DDAC can significantly reduce the current tracking error compared to DDFLC.

### 4.2. Adaptive Controller for the Voltage Loop

DC loads in the AC MG are frequently unknown and time-varying, which places higher requirements on the performance of the voltage controller. This section employs the discrete-time Lyapunov stability theory to design the LPAL for estimation of DC loads in real-time, thereby ensuring the voltage controller’s adaptability to DC loads. The design process is as follows.

Firstly, the proposed discrete-time adaptive voltage controller is as follows:

(27) $$u_{rdc}(k)=\hat{\xi }(k)V_{dc}(k)+C\left[\frac{{V_{dc}}^{*}(k+1)-{V_{dc}}^{*}(k)}{T_{s}}-k_{vdc}e_{u}(k)\right]$$

where $\hat{\xi }(k)$ is the estimated value of *ξ*(*k*).

Secondly, we employ discrete-time Lyapunov theory to design the LPAL. The specific process is as follows.

Define the following Lyapunov function:

(28) $$V_{2}(k)=\frac{1}{2}[Ce_{u}{\left(k\right)}^{2}+\frac{1}{\gamma }{\tilde{\xi }}^{2}(k)]$$

where $\tilde{\xi }(k)$ is the load parameter estimation error, $\tilde{\xi }(k)=\hat{\xi }(k)-\xi (k)$. *γ* is the adaptive gain, *γ* > 0.

**Assumption** **3.** *ξ(k) is slow time-varying, it satisfies ξ(k + 1) = ξ(k). $\tilde{\xi }(k)$ is bounded and satisfies $\tilde{\xi }(k)\leq M_{2}$ , and M_2_ is the upper bound of $\tilde{\xi }(k)$*.

Combining Equations (5) and (27), and Assumption 3, we obtain

(29) $$\begin{array}{cc} \Delta V_{2}(k) & =V_{2}(k+1)-V_{2}(k)\\ & =\frac{1}{2}\left[C{e_{u}}^{2}(k+1)-C{e_{u}}^{2}(k)\right]+\frac{1}{2}\left[\frac{1}{\gamma }{\tilde{\xi }}^{2}(k+1)-\frac{1}{\gamma }{\tilde{\xi }}^{2}(k)\right]\\ & =\frac{1}{2}\left[e_{u}(k+1)+e_{u}(k)\right]\left[Ce_{u}(k+1)-Ce_{u}(k)\right]+\frac{1}{2}\left[\tilde{\xi }(k+1)+\tilde{\xi }(k)\right]\left[\frac{1}{\gamma }\tilde{\xi }(k+1)-\frac{1}{\gamma }\tilde{\xi }(k)\right]\\ & =-CT_{s}k_{vdc}{e_{u}}^{2}(k)+{\left(\left[\frac{\sqrt{C}T_{s}k_{vdc}}{\sqrt{2}}e_{u}(k)]-[\frac{T_{s}}{\sqrt{2C}}\tilde{\xi }(k)V_{dc}(k)\right]\right)}^{2}+\frac{T_{s}}{2}e_{u}(k)V_{dc}(k)[\tilde{\xi }(k)-\tilde{\xi }(k+1)] \end{array}$$

This paper designs the LPAL as follows.

(30) $$\frac{\hat{\xi }(k+1)-\hat{\xi }(k)}{T_{s}}=-\gamma e_{u}(k)V_{dc}(k)$$

Substituting Equation (30) into Equation (29) yields

(31) $$\Delta V_{2}(k)=-CT_{s}k_{vdc}{e_{u}}^{2}(k)+{\left(\left[\frac{\sqrt{C}T_{s}k_{vdc}}{\sqrt{2}}e_{u}(k)]-[\frac{T_{s}}{\sqrt{2C}}\tilde{\xi }(k)V_{dc}(k)\right]\right)}^{2}+\frac{{T_{s}}^{2}}{2}\gamma {e_{u}}^{2}(k){V_{dc}}^{2}(k)$$

According to the Cauchy–Buniakowsky–Schwarz Inequality [28], we obtain

(32) $$\frac{\Delta V_{2}(k)}{{k_{vdc}}^{2}}\leq \frac{-CT_{s}{e_{u}}^{2}(k)}{k_{vdc}}+C{T_{s}}^{2}{e_{u}}^{2}(k)+\frac{{T_{s}}^{2}{V_{dc}}^{2}(k)\gamma }{2{k_{vdc}}^{2}}{e_{u}}^{2}(k)+\frac{{T_{s}}^{2}{V_{dc}}^{2}(k){\tilde{\xi }}^{2}(k)}{C{k_{vdc}}^{2}}$$

Combing this with Assumption 3, if the selected *k_vdc_* satisfies

(33) $$k_{vdc}>>\frac{T_{s}V_{dc}(k)M}{\sqrt{C}}>\frac{T_{s}V_{dc}(k)\tilde{\xi }(k)}{\sqrt{C}}$$

then form Equations (32) and (33); we obtain

(34) $$\Delta V_{2}(k)\leq -CT_{s}k_{vdc}{e_{u}}^{2}(k)+C{T_{s}}^{2}{k_{vdc}}^{2}{e_{u}}^{2}(k)+\frac{{T_{s}}^{2}{V_{dc}}^{2}(k)\gamma }{2}{e_{u}}^{2}(k)$$

Further, if

(35) $$0<\gamma <\frac{2\left(Ck_{vdc}-CT_{s}{k_{vdc}}^{2}\right)}{T_{s}{V_{dc}}^{2}}$$

then Δ*V*_2_ < 0. Combining Equations (9) and (33), *k_vdc_* satisfies

(36) $$\frac{T_{s}V_{dc}(k)M}{\sqrt{C}}<<k_{vdc}<\frac{1}{T_{s}}$$

From the above analysis, it can be concluded that *e_u_*(*k*) and $\tilde{\xi }(k)$ can converge to zero. Further, *M*_2_ can be chosen as a smaller value close to zero. The parameter design procedure for the DDAC is summarized as follows:

Step 1: Use Equations (9) and (36) to select *k_d_*, *k_q_*, and *k_vdc_*. They should be initially set to large values to avoid a slow dynamic response, and then gradually decreased until the system stabilizes and an acceptable dynamic response is achieved.

Step 2: Set the adaptive parameters *λ_d_*, *λ_q_*, and *γ* to large values based on Equations (17) and (35). Adjust these values until the system achieves an optimal steady state and dynamic performance.

**Figure 3:**
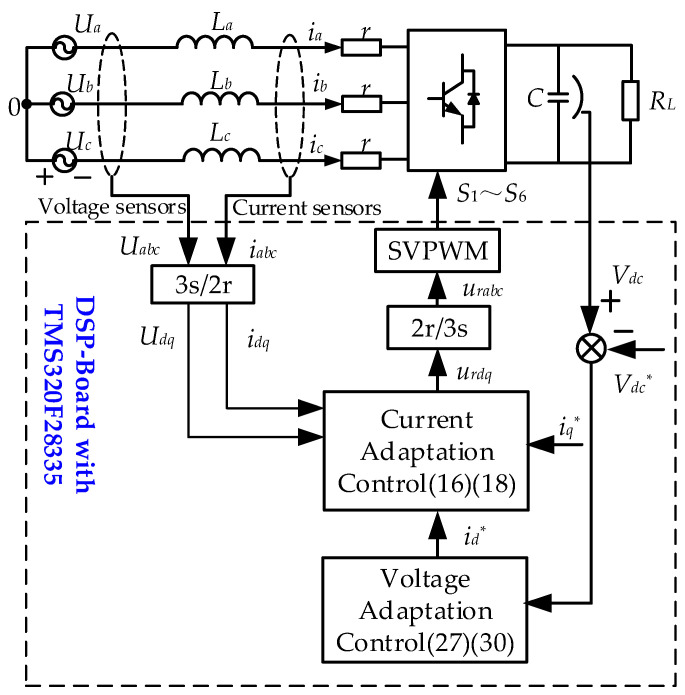
Block diagram of the proposed DDAC.

Based on the above analysis, a block diagram of the DDAC is illustrated in Figure 3.

**Remark** **1.** *Considering the physical properties of inductors and current protection, it is obvious that ΔL, Δr, i_d_(k), and i_q_(k) must be limited. For rectifiers, the sampling frequency is usually high. Related to the sampling system,* *f_s_(k) and ξ(k) can be considered slow time variables, f_s_(k) and ξ(k) are approximated as constants in a sampling period, namely f_s_(k) = f_s_(k + 1), ξ(k + 1) = ξ(k).*

## 5. Simulation and Experimental Verification

**Figure 4:**
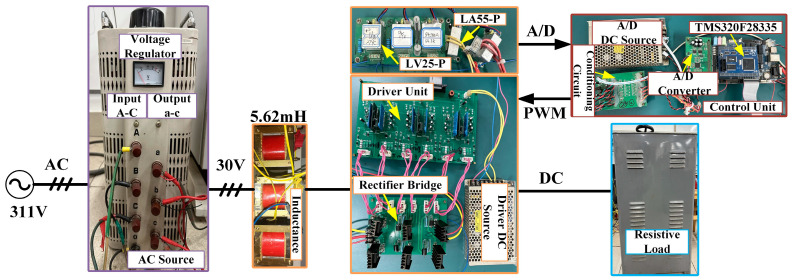
Three-phase PWM rectifier experimental platform.

This paper uses MATLAB/Simulink (2018) for simulations, with the experimental platform shown in Figure 4. In Figure 4, an autotransformer is connected to a 311 V grid to generate 38 V. The voltage and current sensors are LV-25P and LA-55P (LEM Company, Geneva, Switzerland), respectively. The power switching device is IRFP460 (INFINEON Company, Neubiberg, Gemany), and the control algorithm is implemented via TMS320F28335 (TI Company, Dallas, TX, USA). The experimental data are acquired using TPS2024B (Tektronix Inc., Beaverton, OR, USA), TDS1012B-SC (Tektronix Inc.), and DS1204B (RIGOL Company, Suzhou, China). The estimated inductance disturbances and the DC load are obtained from a four-channel DAC7724 (TI Company). A six-channel AD7656 (Analog Devices Company, Wilmington, MA, USA) is selected to collect voltage and current signals. To verify the superiority of the DDAC, experimental and simulation comparisons are conducted with the DDFLC and the DDPIC. The main circuit parameters are listed in Table 1, and the control parameters are listed in Table 2.

### 5.1. Dynamic and Steady-State Performance at Nominal Parameters

**Figure 5:**
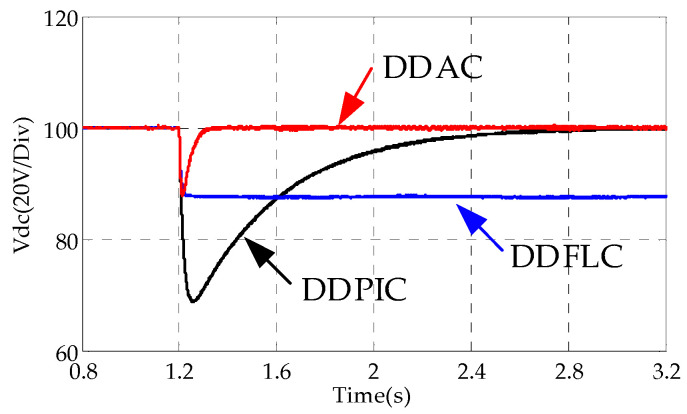
Simulation waveforms when the load steps from 0 Ω to 50 Ω.

**Figure 6:**
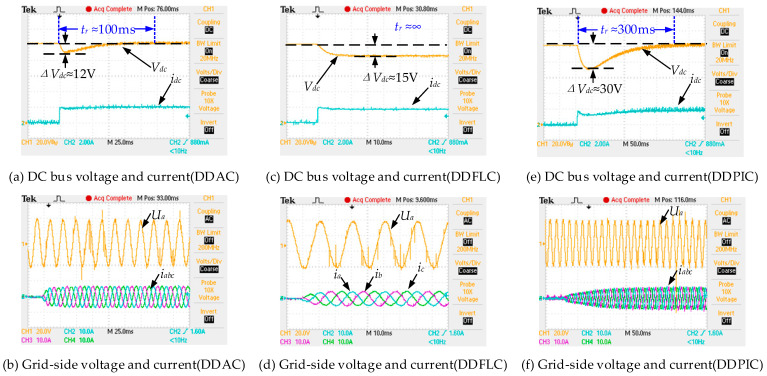
Experimental waveforms when the DC load steps from 0 Ω to 50 Ω.

**Figure 7:**
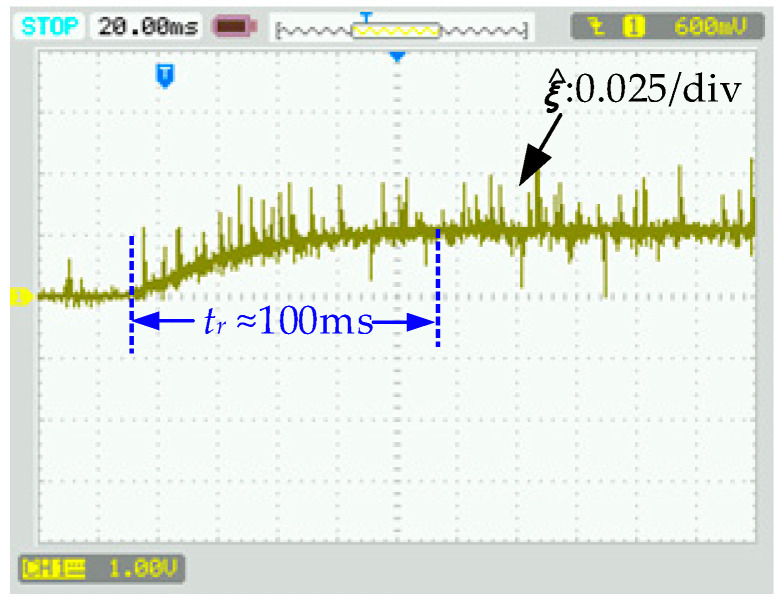
The response of the LPAL when *L*_0_ is equal to *L*.

In this case, the DC load steps up from no load to a load composed of a 50 Ω resistor. Figure 5 and Figure 6 show the transient response of the DC bus voltage. In Figure 5 and Figure 6e, DDPIC exhibits an excellent steady-state performance. However, the PI controller based on the deviation control principle makes it difficult to overcome the control time lag caused by the capacitive element [4], resulting in a low response. From Figure 5 and Figure 6c, it is evident that the DDFLC exhibits a significant steady-state error. This is because increasing the *k_vdc_* can reduce the error, as illustrated in Equation (10). However, a large *k_vdc_* will lead to *V_dc_* instability [15]. Therefore, a compromise *k_vdc_* is selected in this paper. It can be further observed from Figure 5 and Figure 6a,c that, under the same *k_vdc_* condition, the steady state error of DDAC is significantly smaller than that of DDFLC, confirming the correctness of Equations (10) and (26). Figure 7 illustrates the response of the LPAL when *L*_0_ is equal to *L*. It can be seen that $\hat{\xi }(k)$ can quickly and smoothly converge to the steady state value (≈0.025). The theoretically calculated value is about 0.02. This slight discrepancy is attributed to practical factors such as measurement errors, measurement noise, and line impedance. However, this discrepancy does not impact the DC bus voltage tracking effect, as demonstrated in Figure 5 and Figure 6a.

**Figure 8:**
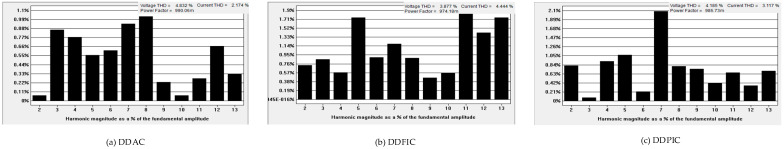
Harmonic analyses under steady-state conditions.

Figure 6b,d,f display the steady-state current waveforms of three control methods. Harmonic analyses with an a-phase current are shown in Figure 8. The current THD values for DDFLC are 4.444% (*i_a_*), 4.383% (*i_b_*), and 4.609% (*i_c_*). The current THD values for DDPIC are 3.117% (*i_a_*), 3.008% (*i_b_*), and 3.396% (*i_c_*), while the current THD values for DDAC are 2.174% (*i_a_*), 2.543% (*i_b_*), and 2.668% (*i_c_*). These results indicate that all methods meet the IEEE 519-2014 standards [29] of a THD below 5%; however, the DDAC exhibits the smallest current THD. As illustrated in Figure 8, the 5th and 7th harmonics under DDAC are smaller than those of DDFLC and DDPIC. Moreover, the 9th, 11th, and 13th harmonics are reduced compared to the DDFLC and the DDPIC. These findings suggest that the DDAC has the best steady-state performance. Additionally, from Figure 8, it is observed that even when the grid voltage THD is high, the DDAC still maintains the minimum current THD. This is because grid voltage harmonics can be considered as a part of *f_s_*(*k*) in Equation (11), and grid voltage harmonics are estimated by the AIDO and compensated for in the DDAC, thereby reducing the influence of grid voltage harmonics on the grid-side current.

### 5.2. Parameter Robustness

**Figure 9:**
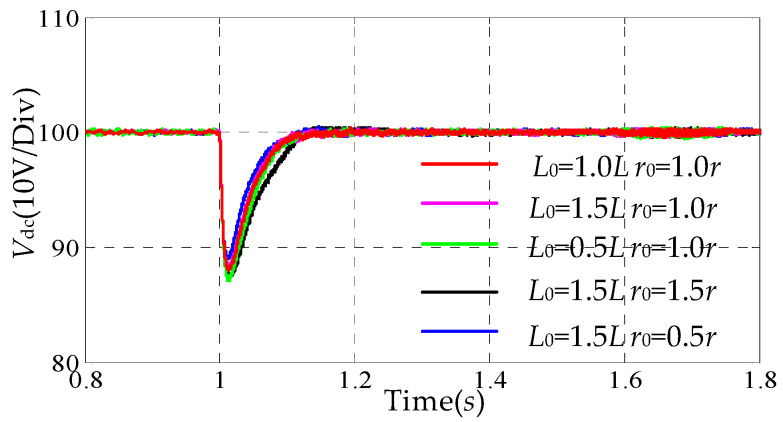
Simulation waveforms under different mismatched inductance parameters.

**Figure 10:**
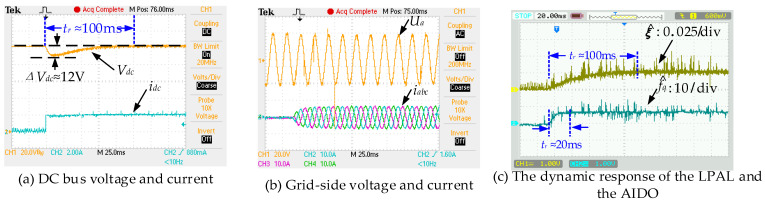
Dynamic responses under mismatched inductance parameters (*L*_0_ = 1.5 *L*, *r*_0_ = 1.0 *r*, load steps from 0 Ω to 50 Ω).

In this section, the robustness of both the DDFLC and the DDAC are investigated under different combinations of *L*_0_ and *r*_0_. It should be noted that the parameters in the control system are mainly changed to evaluate the robustness of the control, because this method can avoid the degradation of filter performance due to the physical changes in its *L* filter and *r* [30]. Figure 9 and Figure 10 show the simulation and experimental results with different combinations of *L*_0_ and *r*_0_. In Figure 9 and Figure 10a, it is observed that the voltage drops and transition times do not change significantly, regardless of how *L*_0_ and *r*_0_ change. These results indicate that the DDAC has a strong robustness to *L* and *r* due to the AIDO compensating for *f_d_*(*k*) and *f_q_*(*k*). Figure 10c shows the transient response of the LPAL and the AIDO when *L*_0_ is equal to 1.5 *L*. It can be seen that $\hat{\xi }(k)$ and ${\hat{f}}_{q}(k)$ can quickly and smoothly transition to steady-state values ($\hat{\xi }(k)$ ≈ 0.025, ${\hat{f}}_{q}(k)$ ≈ 5.3). This indicates that the proposed the LPAL and the AIDO are effective under inductance parameter variations.

**Figure 11:**
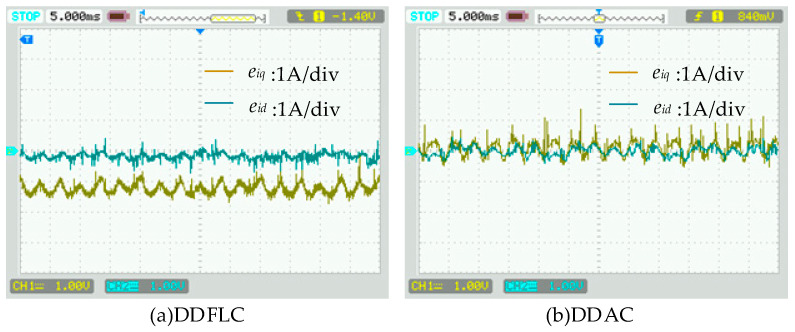
Current tracking errors when *L*_0_ = 1.5 *L*.

Figure 11 shows the current tracking errors of the DDFLC and the DDAC. It can be observed that the current tracking errors with the DDFLC are 0.2 A (d-axis current) and 1 A (q-axis current), while those with the DDAC consistently remain near zero. This indicates that the AIDO significantly enhances the current tracking accuracy of the DDAC, while simultaneously enhancing the DDAC’s robustness to inductance parameters.

## 6. Conclusions

Based on the DDFLC, this paper proposes a DDAC for a three-phase PWM rectifier, which considers inductance-parameter-mismatched and DC load disturbances. An AIDO is designed in the current loop using the gradient descent method to enhance its robustness against inductance-parameter-mismatched disturbances. Additionally, an LPAL is designed in the voltage loop to enable the rectifier system to adapt to load disturbances. The proposed DDAC can be directly employed in digital control systems. Compared with the DDFLC and the DDPIC, in simulation and experiment, the proposed DDAC exhibits the fastest response, the smallest DC bus voltage drop, the smallest current tracking error, and a strong robustness to inductance parameter and load disturbances, while also minimizing its current harmonics contents. The DDAC has the potential to be applied to other converters, such as three-phase three-level Neutral Point Clamped (NPC) rectifiers, thus possessing significant theoretical and engineering value.

## Figures and Tables

**Table 1:** System parameters.

Meaning	Parameters	Value	Units
Grid voltage (peak voltage)	*V_m_*	30	V
Grid frequency	*f*	50	Hz
Filter inductance	*L*	5.62	mH
Equivalent resistance	*r*	1.2	Ω
DC bus reference voltage	*V_dcref_*	100	V
DC filtering capacitor	*C*	1000	μF
Sampling frequency	*f_s_*	9000	Hz

**Table 2:** Parameters of control systems in the experiment.

Controllers	Parameters	Value
DDAC	*k_d_*	50
	*k_q_*	50
	*λ_d_*	10
	*λ_q_*	10
	*k_vdc_*	180
	*γ*	0.00005
DDFLC	*k_d_*	50
	*k_q_*	50
	*k_vdc_*	180
DDPIC	*k_p_d_*	50
	*k_i_d_*	150
	*k_p_q_*	50
	*k_i_q_*	150
	*k_p_vdc_*	180
	*k_i_vdc_*	370

## Data Availability

The data presented in this study are available in this article.
